# Zika Virus Infection, Cambodia, 2010

**DOI:** 10.3201/eid1802.111224

**Published:** 2012-02

**Authors:** Vireak Heang, Chadwick Y. Yasuda, Ly Sovann, Andrew D. Haddow, Amelia P Travassos da Rosa, Robert B. Tesh, Matthew R. Kasper

**Affiliations:** US Naval Medical Research Unit No. 2, Phnom Penh, Cambodia (V. Heang, C.Y. Yasuda);; Ministry of Health, Phnom Penh (L. Sovann);; University of Texas Medical Branch, Galveston, Texas, USA (A.D. Haddow, A.P. Travassos da Rosa, R.B. Tesh);; US Naval Medical Research Unit No. 2, Jakarta, Indonesia (M.R. Kasper)

**Keywords:** Zika virus, viruses, Flavivirus, Cambodia, zoonoses, mosquitoes

**To the Editor:** *Zika virus* (ZIKV), a member of the family *Flaviviridae,* genus *Flavivirus*, was first isolated from the blood of a sentinel rhesus monkey from the Zika Forest of Uganda in 1948 (*1*). Since that time, serologic studies and virus isolations have demonstrated that the virus has a wide geographic distribution, including eastern and western Africa; the Indian subcontinent; Southeast Asia; and most recently, Micronesia (*2**–**5*). The virus is transmitted primarily through the bite of infected mosquitoes and most likely is maintained in a zoonotic cycle involving nonhuman primates (*1*), although recent evidence suggests the possibility of occasional sexual transmission in humans (*4*). Few case reports have described the clinical characteristics of ZIKV infection in humans. Most reports describe a self-limiting febrile illness that could easily be mistaken for another arboviral infection, such as dengue or chikungunya fever. We report a confirmed case of ZIKV infection in Cambodia.

Since 2006, the US Naval Medical Research Unit No. 2 (NAMRU-2) has conducted surveillance for acute fever to determine causes of the infection among patients who seek health care at local clinics in Cambodia. Patients were enrolled by the health clinic physician after they gave informed consent in accordance with an institutional review board protocol approved by NAMRU-2 and the National Ethics Committee for Human Research of Cambodia. At enrollment, the physician administered a questionnaire and collected specimens (blood and throat swabs). All items were transported to the NAMRU-2 laboratory in Phnom Penh, where testing was conducted for a variety of viral, bacterial, and parasitic pathogens. In August 2010, a blood specimen was collected from a 3-year-old boy at a health clinic in Kampong Speu Province, Cambodia. The child’s reported clinical symptoms included 4 days of fever and sore throat and cough and a headache for 3 days. A maculopapular rash was not observed, and the boy was not hospitalized. The clinic staff conducted a follow-up interview and reported that the patient recovered fully.

ZIKV infection was confirmed in this patient by using PCR, sequencing, and serology and through virus isolation. ELISA for chikungunya and dengue virus IgM and IgG antibodies on acute- and convalescent-phase serum was negative. A universal flavivirus real-time PCR screen that targets the nonstructural (NS) 5 gene (*6*) determined that the patient’s serum was positive for flavivirus RNA, but subsequent species-specific PCR ruled out 2 other flaviviruses that are highly endemic to the region (dengue and Japanese encephalitis viruses) (*7**–**9*). This result was the first nondengue, non–Japanese encephalitis virus flavivirus detected after samples from ≈10,000 enrolled patients were tested. Nucleic acid sequencing of the amplicon isolated by gel purification produced a 100-bp fragment with 100% sequence identity to ZIKV (nucleotide position 8,969 of the NS5 gene of the isolate GenBank accession no. EU545988). ZIKV infection subsequently was serologically confirmed by hemagglutination-inhibition tests on paired serum samples. The patient’s acute-phase sample was negative, but a convalescent-phase sample gave a positive reaction with ZIKV antigen to a serum dilution of 1:320 and was negative to antigens for the 4 dengue serotypes and yellow fever and West Nile viruses. These results demonstrate that the patient had a clear monotypic flavivirus immune response with seroconversion against ZIKV, indicating a recent primary infection.

The most common signs and symptoms reported in confirmed ZIKV infections are fever, headache, malaise, maculopapular rash, fatigue or myalgia, and arthritis and arthralgia (Table). In addition to fever and headache, the patient in this study had a sore throat and cough. Because of the patient’s age, additional information about symptoms was difficult to obtain.

**Table:** Reported or observed clinical signs and symptoms in persons with Zika virus infection, 1962–2010

Sign or symptom	Country, year of infection origin,* no. (%) patients					
	Uganda, 1962, n = 1	Laboratory acquired, 1973, n = 1	Indonesia, 1977–1978, n = 7	Micronesia, 2007, n = 28	Senegal/United States, 2009, n = 3	Cambodia, 2010, n = 1
Fever	1 (100)	1 (100)	7 (100)	20 (65)		1 (100)
Headache	1 (100)			14 (45)	3 (100)	1 (100)
Malaise	1 (100)		5 (71)		3 (100)	
Maculopapular rash	1 (100)			28 (100)	3 (100)	
Fatigue or myalgia	1 (100)	1 (100)	1 (14)	14 (45)	1 (33)	
Arthritis and arthralgia			1 (14)	20 (65)	3 (100)	
Chills		1 (100)	2 (29)		2 (67)	
Dizziness			5 (71)			
Joint swelling or edema				6 (19)	2 (67)	
Stomachache			6 (86)			
Retro-orbital pain		1 (100)		12 (39)		
Conjunctivitis			1 (14)	17 (55)	1 (33)	
Anorexia			4 (57)			
Photophobia					1 (33)	
Vomiting			1 (14)	3 (10)		
Diarrhea			3 (43)			
Constipation			3 (43)			
Sore throat						1 (100)
Cough						1 (100)
Aphthous ulcer					2 (67)	
Hypotension			2 (29)			
Hematuria			1 (14)			
Prostatitis					1 (33)	
Hematospermia					1 (33)	
Sweating		1 (100)				
Lightheadedness					1 (33)	

*References: Uganda (*2*), laboratory-acquired (*10*), Indonesia (*5*), Micronesia (*9*), Senegal/United States (*4*). Blank cells indicate no reported information.

The clinical characteristics exhibited by this case-patient are similar to those of shown in a small cluster of ZIKV infections described in Indonesia during 1977–1978 in which maculopapular rash was not observed (*5*). Maculopapular rash was reported as a common sign in case-patients from the recent Yap Island outbreak (*3*), as well as in case reports from Uganda (*2*), Senegal, and the United States (*4*), A case report of laboratory-acquired ZIKV infection also noted the lack of maculopapular rash (*10*).

The clinical features of ZIKV infection are similar to those of dengue virus and chikungunya virus infections, and both arboviruses are found in Southeast Asia. In this region, laboratory-based confirmation is essential. The extent of ZIKV infections in Cambodia is unknown; further studies are needed to clarify the prevalence and geographic distribution of ZIKV infection in the country.
